# Do statins lower testosterone and does it matter?

**DOI:** 10.1186/1741-7015-11-58

**Published:** 2013-02-28

**Authors:** Allan D Sniderman, George Thanassoulis

**Affiliations:** 1 Division of Cardiology, McGill University Health Centre, 687 Pine Avenue West, Montreal, QC H3A 1A1, Canada

**Keywords:** statins, testosterone, drug side effects

## Abstract

Drugs are two-sided swords and statins are no exception. Schooling *et al. *demonstrate that, on average, statins produce small, but statistically significant, decreases in testosterone. They appropriately emphasize that the clinical significance of their observations is unclear but suggest that changes in testosterone might be related to the benefits of therapy as well as the risks, such as the increased chance of diabetes mellitus. Their findings and hypotheses are noteworthy. However, we believe this represents another example of the limitations in the published summaries of drug effects. How do we know all changes induced by drugs are normally distributed? Some may be affected much more than others. Moreover, the confidence intervals of a meta-analysis describe the variance of the mean effect, not the range of effects, and while the mean change characterizes the impact of a drug on a group of patients, the range more fully characterizes its effects on individuals. We treat individuals not groups. Averages do not disclose enough about the risks and benefits of drugs.

See related research article here http://www.biomedcentral.com/1741-7015/11/57

## Background

For so long, statins seemed so simple. All the news, or almost all the news, was good news. However, our confidence, or was it smugness, that we knew all we needed to know about this important class of drugs has ended with the recent evidence that diabetes mellitus may be a side-effect of statin therapy [1], a realization that comes disappointingly late in the game given how many years statins have been used and how many statin studies have been performed; further proof that randomized clinical trials (RCTs) are better tests of the benefits than the risks of therapies and that RCTs are only one element in the full montage of evidence that needs to be assembled about the benefits and risk of drug therapy. The present study by Schooling *et al. *[2] examines another issue that does not seem to have received the attention it deserves - do statins lower testosterone and, if so, could that effect be biologically relevant in terms of how statins achieve their benefit or, perhaps, represent another way how they might harm us [2]?

## Discussion

Cholesterol is an obligate precursor of sex hormones and statins inhibit HMG CoA reductase, the rate-limiting enzyme in the synthesis of cholesterol. In principle, this should not matter as sufficient cholesterol should be delivered to these cells by the low-density lipoprotein (LDL) pathway, the biological alternative to *de novo *synthesis. However, this may represent yet another example of how real life refuses to conform to our models of it, since the meta-analysis by Schooling *et al. *[2] indicates that this does occur. In men, testosterone was lowered by doses of statins they describe as typical (but we would characterize as low to moderate) by about 4% (0.66 mmol/l; 95% CI -0.14 to 1.18) and by about 11% (0.4 mmol/L; 95% CI -0.05 to 0.76) in women.

Five trials with a total of 501 men and six trials with a total of 368 women with polycystic ovary syndrome were analyzed. The decreases were significant for both fixed and random effects models. The authors identify several reasons their results may be less compelling in women. First, funnel plots did suggest some evidence of publication bias, the assays for testosterone may be less reliable in women given the lower levels, and no effect was observed when only the higher quality trials were analyzed. Moreover, the women were selected for a disorder that may be related to abnormal androgen metabolism. By contrast, in men, there was no evidence of publication bias and the results were more homogenous and more robust in that the results in the higher quality studies were the same as in the overall analysis.

This meta-analysis appears to have been well performed with a genuine attempt to identify all the relevant literature as well as an evaluation of factors that might lead to a misleading result. The authors note that statins have been reported to reduce androgens in women with polycystic ovary syndrome and they hypothesize that reduction of androgens might favorably modulate the immune response and reduce atherogenesis. They also hypothesize that lower testosterone levels might be involved in the genesis of the increased risk of diabetes mellitus. However interesting and potentially important these issues are, attention will, doubtless, also focus on whether lower testosterone levels might alter sexual drive and function in men.

Nevertheless, are the findings clinically significant? As the authors fairly point out, a definitive answer is not easy. The average changes are small, the range of normal values for testosterone wide, and there is no clear relation between testosterone concentration and sexual drive and function. Moreover, the clinical information linking statins to sexual dysfunction is extremely limited [3,4]. Accordingly, it would be easy to dismiss the observations as statistically, but not clinically, significant. However, we believe the real lesson to be learned is how inadequately we measure and report the effects of drugs and this directly limits our ability to fully understand their myriad effects.

By convention, the effects of drugs in meta-analysis are expressed as a summary geometric mean and confidence interval. But what evidence supports this convention and why should we assume that it holds for all the effects of a drug? And even if it does, there can be considerable variance in the response to a drug. As illustrated in the JUPITER trial (Justification for the Use of Statins in Primary Prevention: an Intervention Trial Evaluating Rosuvastatin), the lowering of LDL cholesterol (C) by a statin, in this case 20 mg rosuvastatin, is highly variable with 10% of subjects having <20% and 10% >70% decrease in LDL C [5]. The absolute benefit of a statin depends on the absolute lowering of LDL C, which is determined by the initial level of LDL C as well as the potency and dose of the statin [6]. Therefore, the benefits of statins depend on to whom they are given as well as their intrinsic potency. The lesson should be that it is the actual response of a specific patient to an agent that should concern us, not the average response of a group of patients.

This meta-analysis is, necessarily, based on the data as originally reported, which are restricted to the average decrease observed with statins. It is important to appreciate that the 95% confidence intervals reported refer only to variance in the average effect size not, as is commonly thought, to the range of possible outcomes. There is, of course, no *a priori *reason why there might not be substantial dispersion of such decreases in testosterone induced by rosuvastatin just as there is for LDL C lowering, in which case, more extreme changes in plasma testosterone level will occur in an important number of people who take statins. If so, notwithstanding that the average reductions in testosterone in groups of individuals are small, more extreme decreases in testosterone in certain individuals might explain part of the benefits (or risks) of statin therapy. Alternatively, the dispersion may be much less; the effect much more uniform. Our point is that the answer to this 'clinical' question is in the data, the actual data and not the summary calculations of the data.

## Conclusion

In this first meta-analysis of the issue, Schooling *et al. *[2] report that statins lower serum testosterone in both men and women, a potentially important finding. Unfortunately, the clinical implications of this observation are less clear than they could be because the only data available to them were the average changes in testosterone induced by statins. While the average changes may be small, the range of changes in individuals, potentially, might be much more substantial. The problem is that we have become accustomed to expressing the effects of our therapies in terms of the average changes in groups as opposed to also considering the range of changes in individuals. We need to acknowledge this shortcoming if we are going to learn more about the benefits and risks of drugs and really understand whether side effects, as reported by Schooling *et al*., truly matter.

## Abbreviations

C: cholesterol; JUPITER trial: Justification for the Use of Statins in Primary Prevention: an Intervention Trial Evaluating Rosuvastatin; LDL: low-density lipoprotein; RCTs: randomized clinical trials.

## Competing interests

The authors declare that they have no competing interests.

## Authors' contributions

ADS drafted the Commentary. GT critiqued and revised the draft, while ADS prepared the final draft, which they individually reviewed, debated and approved.

## Authors' information

ADS is a senior cardiologist who is interested in learning how to be a better cardiologist and therefore who is, amongst other things, interested in learning how to apply conclusions that hold for the groups that we study to the individuals that we treat. GT is a cardiologist and genetic epidemiologist who is interested in the prevention of cardiovascular disease with a particular focus in valvular heart disease.

## Pre-publication history

The pre-publication history for this paper can be accessed here:

http://www.biomedcentral.com/1741-7015/11/58/prepub
